# Cytomegalovirus Colitis in Adult Patients with Inflammatory Bowel Disease

**DOI:** 10.3390/v17060752

**Published:** 2025-05-24

**Authors:** Kriti Soni, Alfredo Puing

**Affiliations:** 1 Government Medical College and Hospital, Chandigarh 160047, India; kritisoni50@gmail.com; 2 Division of Infectious Diseases, Yale University, New Haven, CT 06510, USA

**Keywords:** cytomegalovirus, inflammatory bowel disease, colitis

## Abstract

Cytomegalovirus (CMV) colitis, a complication in patients with inflammatory bowel disease (IBD), particularly ulcerative colitis (UC), is a significant diagnostic and therapeutic challenge due to its overlap with IBD flares. CMV reactivation in IBD is driven by chronic inflammation, compromised immune function, and use of immunosuppressive agents like corticosteroids. Risk factors include older age, pancolitis, and severe disease. Diagnosis hinges on endoscopy and histology, with tissue biopsy and immunohistochemistry as the gold standard. Quantitative tissue PCR may aid in differentiating latent from active infection. CMV colitis exacerbates IBD symptoms, prolongs hospitalization, and increases colectomy rates. Antiviral therapy, primarily ganciclovir, improves outcomes in patients with corticosteroid-refractory UC. Treatment focuses on tapering corticosteroids, optimizing biologic therapies such as infliximab, and a careful application of antivirals tailored to disease severity and viral load. Further research is needed to refine diagnostic thresholds and treatment strategies to mitigate CMV’s impact on IBD prognosis. Early identification and individualized management are critical to improving clinical outcomes and reducing morbidity.

## 1. Introduction

Cytomegalovirus (CMV) is a ubiquitous double-stranded DNA virus belonging to the β-herpesviridae subgroup of the Herpesviridae family. Human CMV infection is very common, and most humans become infected during childhood. In the United States, CMV seroprevalence among adults is estimated to be 50%; however, this rate varies depending on age, geography, and socioeconomic status [1]. After CMV infection, CMV establishes a lifelong latent infection in CD34+ hematopoietic progenitor cells, CD14+ monocytes, macrophages, and dendritic cells. CMV infection can exist in a latent state, characterized by the presence of CMV viral DNA without active replication, or in an active state, marked by detectable viral replication, often without accompanying symptoms. During latency, CMV maintains a quiescent state with minimal viral gene expression, which helps it evade the host immune surveillance [2]. Primary CMV infection is often asymptomatic or can present as a mononucleosis-like syndrome in immunocompetent hosts. In contrast, CMV disease refers to the presence of clinical symptoms directly associated with active CMV infection, particularly in immunocompromised hosts. Clinical syndromes that may be observed in this setting include encephalitis, pneumonitis, hepatitis, uveitis, retinitis, colitis, and graft rejection [3,4].

CMV infection is more prevalent in patients with inflammatory bowel disease (IBD) than in the general population, with several factors increasing their susceptibility to latent CMV reactivation. These include ongoing inflammation in the colon, poor nutritional status, compromised natural killer cell functionality, and the use of long-term immunosuppressive therapy for maintenance treatment [5]. Furthermore, the development of CMV colitis poses significant challenges in the management of IBD, particularly ulcerative colitis (UC). It usually presents with a combination of gastrointestinal and systemic symptoms that mimic an IBD flare, including bloody diarrhea, abdominal pain, fever, and weight loss. In some cases, severe complications such as toxic megacolon, colonic perforation, and death can occur. Additionally, CMV colitis can exacerbate UC symptoms, complicating the clinical picture and treatment course, particularly in patients with steroid-refractory UC, where symptoms persist despite immunosuppressive therapy. Other CMV end-organ damage is not commonly seen in IBD [6]. Here, we aim to review the latest evidence on the pathophysiology, diagnosis, and management of CMV infection in patients with IBD.

## 2. Risk Factors Contributing to CMV Colitis

Several risk factors and clinical indicators for CMV colitis in IBD patients have been identified, including demographics, patient-specific factors, and laboratory findings.

### 2.1. Demographic Factors

Females and older individuals have been shown to be more susceptible to CMV infection, with age consistently emerging as a risk factor for CMV reactivation [7,8,9]. This is further supported by a recent meta-analysis, which found that UC patients with a later age of disease onset are more likely to experience CMV reactivation [10].

### 2.2. Disease-Specific Factors

Disease severity and extent are other key risk factors for CMV colitis with pancolitis nearly doubling the risk of CMV infection compared to those with lesions confined to the left colon [11]. Similarly, a meta-analysis by Qin et al. found that the risk of CMV reactivation in severe UC was 1.5 times higher than in mild-to-moderate cases, and Lee et al. reported a 1.5-fold increased risk of CMV infection for each point increase in the Mayo score in patients with acute severe colitis [10,12].

### 2.3. Patient-Specific Factors and Laboratory Findings

Patient-specific risk factors include exposure to antibiotics, hypoalbuminemia, and elevated creatinine levels [8,13]. Clinically, CMV colitis is often linked to anemia, widespread colonic involvement during colonoscopy, and the presence of ulcers observed during endoscopy [9]. Additionally, reduced hemoglobin and elevated C-reactive protein levels are key laboratory predictors of CMV colitis [14].

### 2.4. Use of Immunosuppressants

The use of immunosuppressants also has a significant role in CMV reactivation and disease. Corticosteroids have been shown to increase the risk of CMV reactivation by fourfold. Lee et al. [6] reported that an average daily glucocorticoid dose exceeding 40 mg within one month significantly raises the likelihood of CMV reactivation. Furthermore, other studies have identified a cumulative glucocorticoid dose of more than 400 mg over a four-week period as a critical risk factor. Interestingly, 5-ASA use has been linked with a lower risk of CMV reactivation, possibly because treated patients have a milder disease and a subsequently lower incidence of CMV reactivation. A comprehensive analysis examining 2099 individuals revealed a notable link between CMV reactivation in UC patients and factors such as pancolitis, advanced age, and the use of immunosuppressants, azathioprine, and steroids [10,12].

Table 1 shows risk factors for CMV colitis in patients with IBD.

## 3. Pathophysiology

IBD mainly encompasses two primary conditions: Crohn’s disease (CD) and ulcerative colitis (UC). Anatomically, CD and UC affect the digestive tract differently. In UC, the rectum is consistently involved, along with continuous lesions in varying extents of the colon, whereas in CD, the lesions are patchy and discontinuous, occurring throughout the entire digestive tract. They are clinically characterized by alternating acute inflammatory flares and asymptomatic phases during remission [15]. In these patients, CMV seroprevalence is not well described but is assumed to be comparable to 50% of the general population in the United States [1]. CMV-seropositive individuals carry the latent CMV genome in various tissues, including the entire digestive tract, with the colon being a significant site for both viral latency and reactivation [16]. In the setting of chronic inflammation due to IBD, studies have shown that pro-inflammatory cytokines like tumor necrosis factor-alpha (TNF-α) and interleukin-6 (IL-6) play a significant role in promoting CMV replication in seropositive patients. TNF-α, in particular, can stimulate CMV reactivation by activating the immediate early genes of the virus, which are critical for viral replication. Additionally, IL-6 contributes to CMV-related inflammation and further compromises cellular barriers, leading to viral spread and sustained inflammation in affected tissues, particularly in the gastrointestinal tract. These interactions create a vicious cycle of infection and inflammation [17,18]. However, it is worth noticing that the immune response in IBD varies between CD and UC. UC is associated with a TH2 cytokine profile, which does not effectively prevent CMV replication, whereas CD is associated with a TH1/TH17 profile that can inhibit CMV replication through the production of interferon-gamma (IFN-γ) [19]. This difference in immune response partially explains why CMV reactivation is more common in UC than in CD.

CMV colitis tends to develop in inflamed or ulcerated areas, triggered by mucosal damage and local inflammation. Furthermore, IBD patients receiving immunosuppressive therapy are exposed to both inflammation and weakened immune defense, two key factors that promote CMV reactivation [16]. Corticosteroids, which are commonly used as the first line of treatment for moderate to severe IBD flare-ups, have been shown to promote CMV reactivation by downregulating the activity of monocytes and T-lymphocytes, which are critical in controlling latent viral infections. Additionally, corticosteroids facilitate the transcription of CMV’s immediate early genes, promoting viral reactivation in infected cells [20,21]. Thiopurines promote apoptosis in T-lymphocytes by altering intercellular signaling pathways and have been shown to impair the function of CMV-specific T-lymphocytes and natural killer cells [22]. A recent meta-analysis involving 16 observational studies found that corticosteroid exposure in IBD patients doubled the risk of CMV reactivation in tissues, with an odds ratio (OR) of 2.10 and a 95% confidence interval (CI) of 1.31–3.37. In the same study, patients with UC treated with thiopurines also saw an increase in the risk of CMV infection [23].

## 4. Clinical Features

The clinical presentation of CMV infection in IBD patients can be diverse and often overlaps with symptoms of an IBD flare-up, making diagnosis particularly challenging [24]. Common manifestations include persistent or worsening abdominal pain, diarrhea, and rectal bleeding, which may be accompanied by systemic symptoms such as fever, weight loss, and fatigue. These symptoms can be indistinguishable from those of an IBD exacerbation, leading to potential delays in diagnosis and appropriate treatment. In severe cases, patients may develop life-threatening complications such as toxic megacolon or intestinal perforation, which require immediate medical intervention [25]. In addition, CMV infection can exacerbate the underlying IBD, leading to increased disease severity and resistance to conventional IBD treatments. This synergistic effect between CMV and IBD can result in a more aggressive disease course, prolonged hospitalization, and increased morbidity [26].

Endoscopic findings in CMV-infected IBD patients may reveal extensive ulcerations, with characteristic colonoscopic features of CMV-associated colitis including deep ulcers, punched-out ulcers, geographical ulcers, longitudinal ulcers, and mucosal defects [11]. Univariate logistic regression analysis revealed that severe easy bleeding was seen more frequently in CMV-positive patients than in CMV-negative patients (OR = 2.20, 95% CI: 1.14–4.28). Wide mucosal defects (OR = 4.58, 95% CI: 2.21–10.73), punched-out ulcerations (OR = 3.39, 95% CI: 1.78–7.46), longitudinal ulcerations (OR = 3.09, 95% CI: 1.66–6.26), and a cobblestone-like appearance (OR = 2.05, 95% CI: 1.11–3.82) were more frequently observed in CMV-positive patients than in CMV-negative patients [27].

The impact of CMV infection on long-term outcomes in IBD remains an area of active research, with evidence suggesting that CMV infection significantly correlates with poorer outcomes, such as prolonged hospitalization, colectomy, and increased mortality [12,28,29]. A meta-analysis further emphasizes the strong association between CMV infection and adverse IBD prognosis. Given its potential to complicate management and worsen outcomes, a high index of suspicion for CMV infection is essential in IBD patients with refractory or worsening symptoms as early recognition and appropriate treatment of CMV infection in this population can improve clinical outcomes and help prevent complications [30].

## 5. Diagnosis

A prompt and accurate diagnosis of CMV reactivation is crucial, especially in high-risk IBD patients, as it is a potentially reversible condition linked to poor clinical outcomes, particularly in corticosteroid-refractory UC [31]. Differentiating between an acute UC flare and CMV colitis can be challenging, as both conditions present with similar clinical symptoms, including fever, malaise, diarrhea, hematochezia, abdominal pain, and weight loss. While endoscopic findings like punched-out ulcers may suggest CMV colitis, no endoscopic feature is definitively pathognomonic for distinguishing between the two conditions [32,33]. Another critical distinction is between CMV infection and CMV disease. CMV infection, which may be detected through CMV serology, serum antigenemia, or positive polymerase chain reaction (PCR), does not always translate into active CMV disease. Active CMV disease is defined by the presence of symptoms or CMV-related tissue damage, and studies have shown that these noninvasive tests often correlate poorly with active disease [34,35].

**Figure 1:**
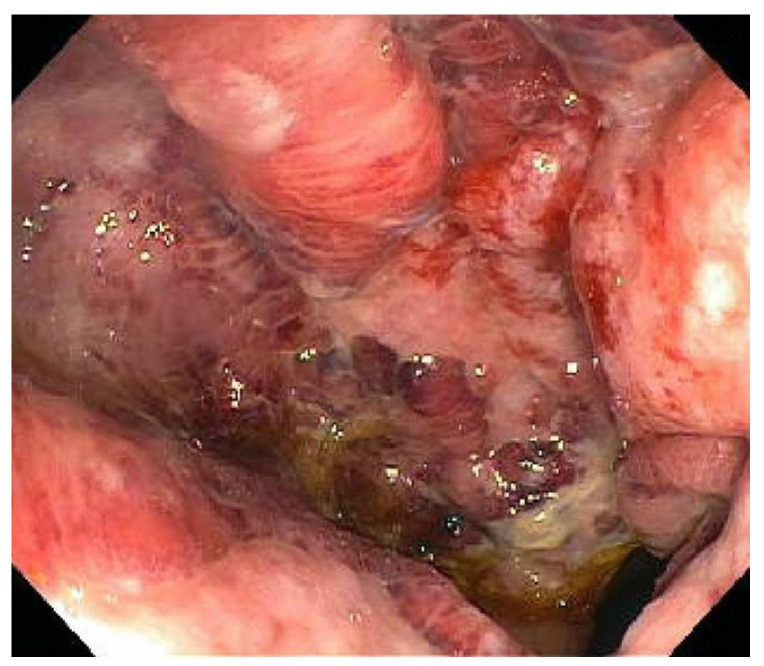
Colonoscopy image showing mucosal ulceration of the distal rectum in CMV colitis in an immunocompetent patient [36].

**Figure 2:**
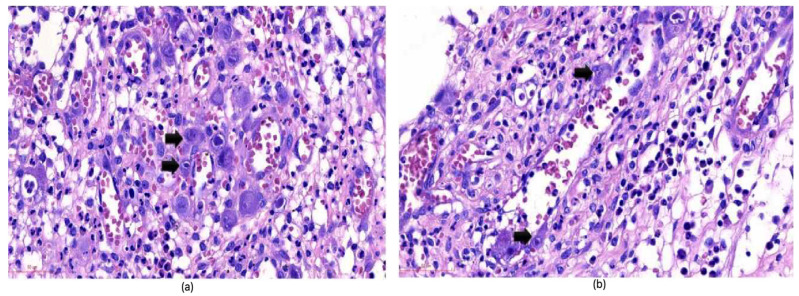
CMV-infected (**a**) mesenchymal cells from colonic tissue; (**b**) endothelial cells from colonic tissue. These CMV-infected cells have large ovoid nuclei with basophilic intranuclear inclusions (Cowdry bodies) surrounded by a clear halo (arrows) [37].

The diagnosis of CMV colitis generally requires a combination of clinical evaluation, diagnostic testing, and a high index of suspicion for accurate identification. Endoscopic examination and histological analysis are crucial, and immunohistochemistry (IHC), along with tissue PCR, are key to confirming active CMV colitis in IBD patients and should be regarded as gold standard diagnostic tests [6]. Endoscopic findings suggestive of CMV colitis include ulcerations, erosions, and mucosal inflammation (Figure 1). Histological examination may reveal characteristic “owl’s eye” inclusion bodies, which are pathognomonic for CMV infection (Figure 2). Left-colon biopsies identify most UC patients with CMV. Conversely, in CD, many patients had CMV detectable only in right-colon biopsies. A minimum of 11 biopsies for UC and 16 biopsies for CD was proposed in a study by McCurdy et al. to achieve an 80% probability of CMV detection [33]. The clinical significance of a positive PCR result for CMV DNA in colonic tissue without accompanying histological signs of infection is not well defined. The detection of viral DNA in the absence of histological evidence of infection is often interpreted as a sign of low-level reactivation or latent CMV infection. Consequently, it has been recommended that quantitative rather than qualitative PCR should be used, as higher viral loads are more closely associated with active CMV colitis and may predict better response to antiviral therapy.

Viral culture, once considered the gold standard for CMV detection, is now obsolete in routine clinical practice due to limited sensitivity and its time-consuming nature. This method involves isolating the virus from tissue samples and growing it in cell culture. While it offers high specificity, the sensitivity of viral culture can be lower compared to more modern techniques like PCR. The main advantage of viral culture is its ability to detect viable, replication-competent viruses, which directly indicates active infection. However, the process typically takes 1–3 weeks to yield results, which may delay diagnosis and treatment initiation. Its clinical utility today is minimal, and CMV culture is no longer commercially available [3,38]. Furthermore, while viral culture was historically used to assess antiviral resistance through phenotypic testing, this is no longer a standard approach, as current practice favors genotypic testing [39].

**Figure 3:**
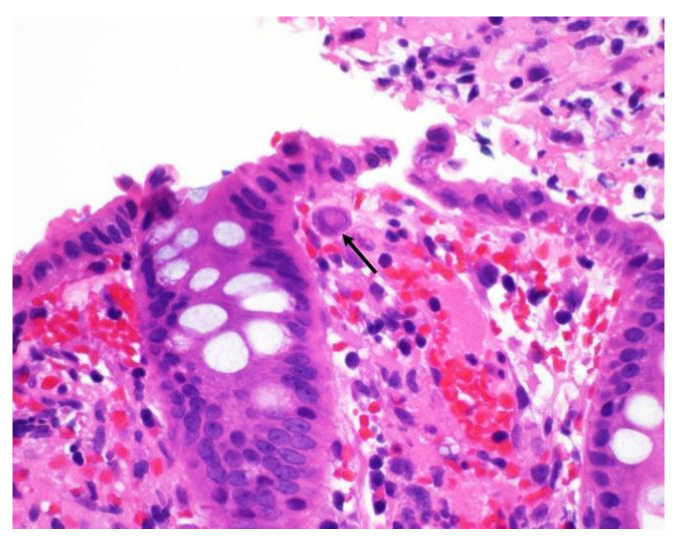
H&E stain of rectal biopsy, showing a large nucleus (arrow) with a smudged, eosinophilic chromatin pattern consistent with cytomegalovirus colitis [36].

**Figure 4:**
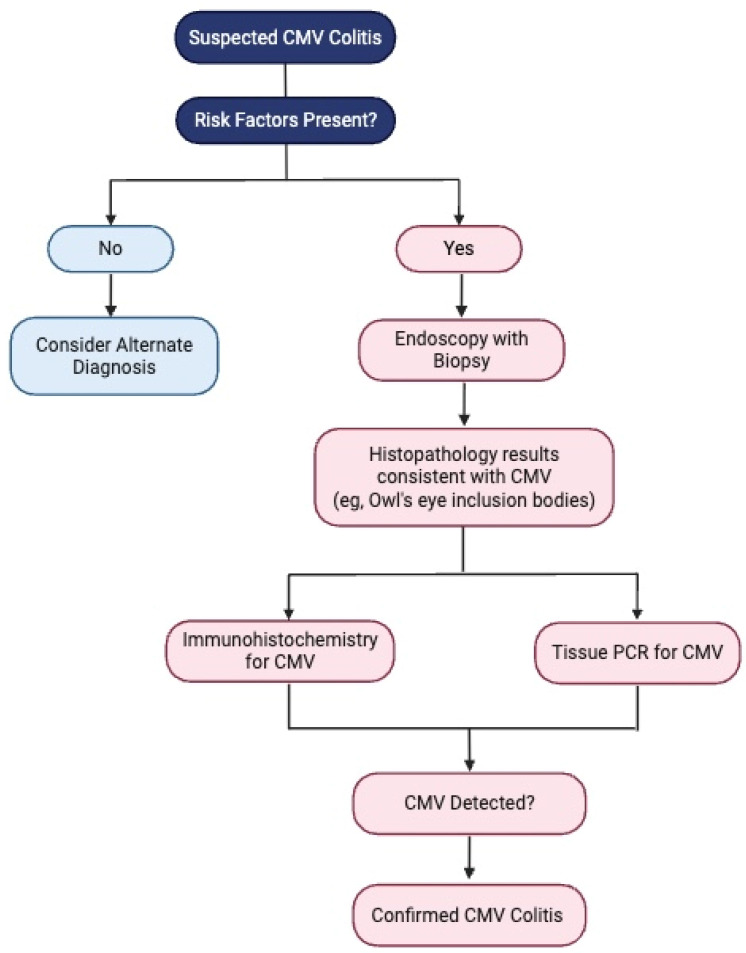
Diagnostic approach for CMV colitis.

Noninvasive diagnostics, such as whole-blood PCR and pp65 antigenemia, are frequently used to assess systemic CMV viremia, especially in immunocompromised patients like transplant recipients, where they help guide pre-emptive antiviral therapy. However, their utility in predicting CMV colitis in IBD patients remains uncertain, as blood-based PCR detection does not always correlate with tissue-invasive disease in the colon. Studies have shown that while these tests are highly specific, they lack the sensitivity required to reliably detect colonic CMV reactivation in IBD patients [32,33]. Blood-based tests, including PCR, have a high positive predictive value (PPV), which can make a positive result useful in reducing the need for invasive endoscopic procedures. However, transient CMV viremia, which may not require treatment, complicates the interpretation of these tests. It remains unclear whether blood-based tests can accurately predict the viral burden in colonic mucosa, which is crucial for understanding disease severity and response to antiviral therapy [35]. Given these limitations, tissue biopsy remains the gold standard for diagnosing CMV colitis, particularly in patients with moderate-to-severe IBD, where early identification and treatment can significantly influence outcomes (Figure 3). Blood-based tests can serve as a valuable adjunct in determining when to withhold immunosuppressive therapy or in predicting the risk of colectomy, but they should not replace tissue-based diagnostics [34]. A summary of the investigations and of the diagnostic approach required for diagnosing CMV colitis is provided in Table 2 and Figure 4.

## 6. Treatment Strategies for CMV in UC Patients

The treatment of CMV infections in IBD patients depends on the severity of the infection and the patient’s immunosuppressive status. CMV can cause IBD exacerbations, especially in patients with corticosteroid-refractory UC [31]. CMV DNAemia is not unusual in IBD patients receiving immunosuppression, and low-level reactivation may disappear without antiviral treatment [40]. Conversely, when CMV is found in the colonic tissue of high-risk patients, it is linked to a higher risk of colectomy, death, and increased healthcare use, suggesting it may play a role in worsening the disease [41,42]. In cases of high viral load or severe inflammation, including deep colonic ulcers, antiviral treatment has been shown to improve outcomes in patients who have not responded to immunosuppressive therapies [43]. A meta-analysis found that antiviral therapy reduced the risk of colectomy by 80% in CMV-positive UC patients who were resistant to corticosteroids, emphasizing the importance of early diagnosis and timely antiviral treatment in high-risk cases [44].

### 6.1. Role of Immunosuppressants and Biologics

Upon diagnosing CMV colitis, the focus should shift to gradually tapering corticosteroids, and its use should be reconsidered only once CMV infection has been properly treated. There is limited direct evidence on the optimal timing for initiation of biological therapies in this setting. However, an alternative anti-inflammatory should be introduced to promote remission. Infliximab is generally preferred over ciclosporin when feasible, given the potential TNF-avidity of CMV [45]. In a case series involving 23 patients, Minami et al. reported association between ciclosporin treatment and increasing likelihood for CMV reactivation [46]. Conversely, monoclonal antibodies targeting TNF-alpha, such as infliximab and adalimumab, have not been found to increase the risk of CMV reactivation. Studies show that CMV tissue infection is not associated with clinical resistance to these therapies. The beneficial effects of anti-TNF-α therapies on CMV infection are likely due to the reduction in TNF-α pro-inflammatory actions, which can otherwise promote viral replication. As a result, these biotherapies are recommended for managing moderate to severe flare-ups of IBD, particularly in cases complicated by CMV colonic infection [10,16].

Additionally, thiopurines should be discontinued, at least temporarily [23]. Vedolizumab is a monoclonal therapeutic antibody that is an antagonist to α4β7 integrin which is expressed specifically by gastrointestinal-homing T lymphocytes. This unique gut selectivity by Vedolizumab offers a more beneficial patient safety profile as compared to other biologics that have action on multiple targets to reduce inflammation [47]. Vedolizumab is generally regarded as a safe biologic when co-administered with antivirals [48,49,50], with a case reporting development of CMV colitis while on Vedolizumab [51]. However, its potential systemic effects warrant caution, and its use should be carefully considered in individuals with CMV colitis.

Studies evaluating safety of Tofacitinib in IBD patients reported rare occurrences of CMV colitis [52,53]. Ustekinumab was linked to a favorable safety profile in a pooled safety analysis of phase 2/3 studies, with two cases of CMV colitis in UC patients, although they were receiving concomitant corticosteroids [54].

### 6.2. Antiviral Therapy

Guidelines differ on the timing of antiviral therapy initiation in IBD patients with CMV infection. Both the American College of Gastroenterology (ACG) and the European Crohn’s and Colitis Organisation (ECCO) recommend starting antiviral treatment in cases of moderate-to-severe colitis when histology reveals a high density of CMV in mucosal tissue and in those who are corticosteroid-refractory or corticosteroid-dependent [55]. There are limited data on the correlation between the progression of UC and tissue viral load, as assessed by viral inclusions through IHC or CMV DNA copy numbers. Some studies have shown that a higher colonic viral load correlates with an increased risk of colectomy, suggesting the potential benefit of antiviral therapy for CMV reactivation in UC patients [41,56]. However, the precise threshold for determining which patients may benefit from antiviral treatment remains undefined. Although conclusive data on the use, mode of administration, and duration of antiviral treatment in CMV colitis are lacking, the ECCO, based on extensive experience with stem cell and solid organ transplant recipients, suggests using intravenous ganciclovir at a standard dose of 5 mg/kg every 12 h. If patients respond within 3 to 5 days, they can be transitioned to oral valganciclovir, generally at a dose of 900 mg twice daily. Treatment duration is individualized based on clinical and virologic response, with a minimum course of 2 weeks, extendable to 6 weeks or longer if necessary. For patients with a detectable viral load, weekly blood PCR testing can help monitor and guide ongoing therapy until the DNAemia is cleared. In cases without measurable viral load, symptom improvement—particularly in diarrhea—serves as the primary indicator of treatment response. If symptoms persist, repeat endoscopic evaluation may be needed. Myelosuppression is the most common severe side effect which requires weekly blood counts. G-CSF support is uncommon but may be necessary. Dose adjustments are crucial for those with renal impairment to avoid overdosing, which can cause drug toxicity, as well as underdosing, which can lead to treatment failure and potential resistance. Foscarnet may be used for patients intolerant to ganciclovir or in rare cases of ganciclovir-resistant CMV. Strict monitoring of renal function and electrolytes is essential. Concomitant administration of normal saline can help reduce the risk of irreversible renal damage. High concentrations of the drug are excreted in the urine, which may cause significant irritation and ulceration in the genital area. Maintaining careful hygiene can mitigate this risk [6].

**Figure 5:**
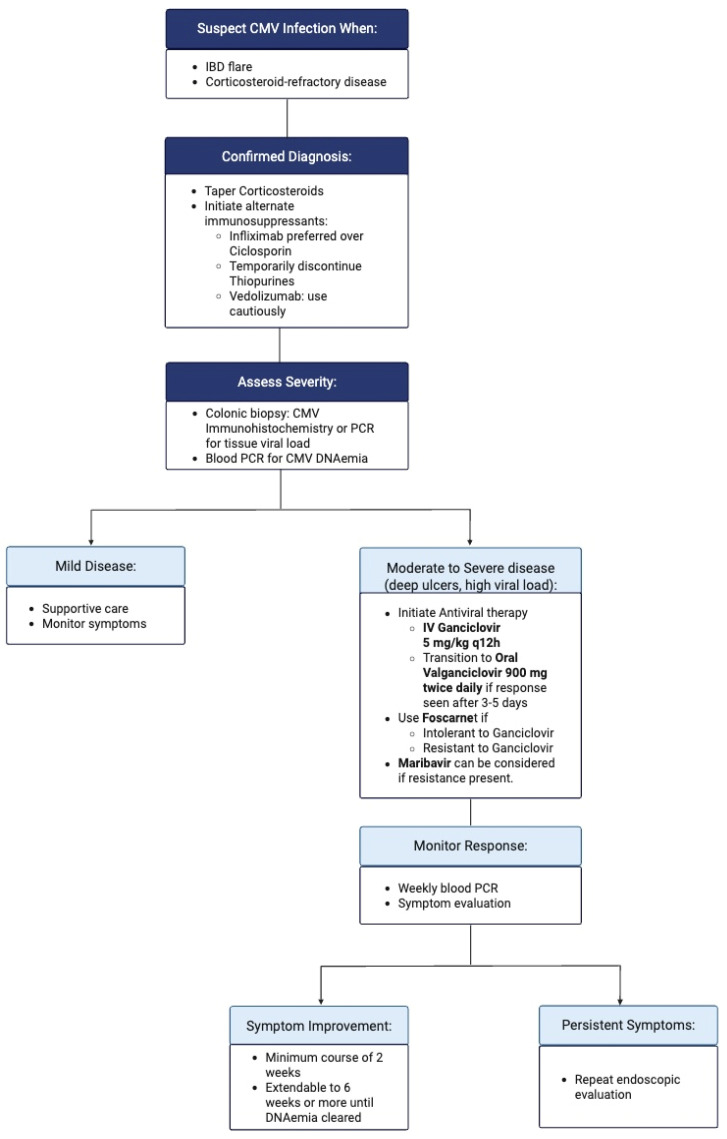
Treatment approach for CMV colitis.

Maribavir, a viral U97 kinase inhibitor, has been FDA-approved for the treatment of refractory or resistant CMV infection [57]. However, there is lack of significant clinical experience with Maribavir in the setting of IBD-associated CMV colitis, and its role in this context remains to be defined. Figure 5 shows a schematic representation of treatment approach for CMV colitis.

### 6.3. Prophylaxis

While antiviral prophylaxis is used routinely in transplant recipients, there is little evidence to guide primary or secondary prophylaxis in patients with IBD; furthermore, the potential for adverse events does not justify standard chemoprophylaxis [58].

## 7. Prognosis and Negative Prognostic Factors

Patients’ prognosis in CMV colitis is determined by multiple factors. CMV infection is negatively influenced by prolonged hospitalization, enhanced need for colectomy, and higher mortality rate [12,28,29]. In a study by Melotti et al., CMV DNAemia was shown to be a strong predictor of colonic CMV involvement and a negative prognostic marker, with the colectomy rate reaching 54.1% in DNAemia-positive patients compared to 34.4% in those without it [12].

Negative prognostic factors include the following:

- High colonic viral load (detected via immunohistochemistry or quantitative PCR).
- Extensive or deep ulcerations on endoscopy [11].
- Systemic inflammatory response—severe anemia, hypoalbuminemia, and elevated CRP levels [14].
- Older age and greater comorbidity burden, as measured by the Charlson Comorbidity Index [10,12].
- Corticosteroid refractory disease.

## 8. Conclusions

Cytomegalovirus (CMV) colitis represents a significant clinical challenge in patients with inflammatory bowel disease (IBD), particularly in those with ulcerative colitis (UC). Its overlapping presentation with IBD flares complicates a timely diagnosis and effective management. Accurate diagnosis relies on a combination of clinical evaluation and histological analysis, with tissue biopsy remaining the gold standard. Early identification of CMV infection in high-risk patients, particularly those with corticosteroid-refractory UC, allows for the initiation of targeted antiviral therapy, which has been shown to improve outcomes and reduce the need for colectomy. Treatment strategies should prioritize the tapering of corticosteroids, selective use of biologics, and the judicious application of antiviral agents such as ganciclovir or valganciclovir. Adopting individualized treatment plans based on disease severity, viral load, and patient response can optimize clinical outcomes while minimizing complications.

Further research is needed to refine diagnostic thresholds, define optimal antiviral regimens, and explore the role of biologic therapies in managing IBD complicated by CMV colitis. Clinicians must maintain a high index of suspicion for CMV colitis in patients with refractory or severe IBD symptoms to ensure timely and effective management, ultimately improving patient outcomes and quality of care.

## Figures and Tables

**Table 1:** Key risk factors for cytomegalovirus (CMV) colitis in patients with inflammatory bowel disease (IBD).

Category	Risk Factors
Demographic factors	Older age Female sex
Disease severity	Severe UC Pancolitis High Mayo score
Clinical features	Persistent anemia Hypoalbuminemia Elevated creatinine Elevated CRP levels
Immunosuppressants	Corticosteroid use (>40 mg/day or cumulative dose > 400 mg in 4 weeks) Long term immunosuppressant use Azathioprine or Thiopurine therapy
Colonoscopy findings	Widespread colonic ulcerations
Other risk factors	Antibiotic exposure Poor nutritional status

**Table 2:** Investigations for the diagnosis of CMV colitis in patients with IBD.

Investigation	Findings	Comments
Endoscopy	Punched-out ulcers, erosions, mucosal inflammation	Unable to distinguish between CMV colitis and IBD flare
Histology	Owl’s eye inclusions	Pathognomonic, gold standard
Immunohistochemistry (IHC)	CMV antigens in tissues	Sensitive and specific, gold standard
Tissue PCR	CMV DNA in tissues	Quantitative PCR preferred, high viral load correlated with active disease
Whole-blood PCR and pp65 antigenemia	CMV DNA and pp65 antigen in blood	Assesses systemic viremia, poor correlation with CMV colitis
Viral culture	Grows viable virus	High specificity, low sensitivity, slow turnaround time

## Data Availability

Not applicable.

## Abbreviations

The following abbreviations are used in this manuscript:

ACG	American College of Gastroenterology
CI	Confidence Interval
CD	Crohn’s Disease
CMV	Cytomegalovirus
ECCO	European Crohn’s and Colitis Organisation
G-CSF	Granulocyte-Colony Stimulating Factor
IHC	Immunohistochemistry
IBD	Inflammatory Bowel Disease
IFN-ɣ	Interferon-gamma
IL-6	Interleukin-6
OR	Odds Ratio
PCR	Polymerase Chain Reaction
PPV	Positive Predictive Value
TNF-ɑ	Tumor Necrosis Factor-alpha
UC	Ulcerative Colitis
